# Contrasting seasonal drivers of virus abundance and production in the North Pacific Ocean

**DOI:** 10.1371/journal.pone.0184371

**Published:** 2017-09-07

**Authors:** P. Jackson Gainer, Helena L. Pound, Alyse A. Larkin, Gary R. LeCleir, Jennifer M. DeBruyn, Erik R. Zinser, Zackary I. Johnson, Steven W. Wilhelm

**Affiliations:** 1 Department of Microbiology, The University of Tennessee, Knoxville, TN, United States of America; 2 Nicholas School of the Environment and Biology Department, Duke University Marine Laboratory, Beaufort, NC, United States of America; 3 Biosystems Engineering & Soil Sciences, The University of Tennessee, Knoxville, TN, United States of America; Oklahoma State University, UNITED STATES

## Abstract

The North Pacific Ocean (between approximately 0°N and 50°N) contains the largest continuous ecosystem on Earth. This region plays a vital role in the cycling of globally important nutrients as well as carbon. Although the microbial communities in this region have been assessed, the dynamics of viruses (abundances and production rates) remains understudied. To address this gap, scientific cruises during the winter and summer seasons (2013) covered the North Pacific basin to determine factors that may drive virus abundances and production rates. Along with information on virus particle abundance and production, we collected a spectrum of oceanographic metrics as well as information on microbial diversity. The data suggest that both biotic and abiotic factors affect the distribution of virus particles. Factors influencing virus dynamics did not vary greatly between seasons, although the abundance of viruses was almost an order of magnitude greater in the summer. When considered in the context of microbial community structure, our observations suggest that members of the bacterial phyla Proteobacteria, Planctomycetes, and Bacteroidetes were correlated to both virus abundances and virus production rates: these phyla have been shown to be enriched in particle associated communities. The findings suggest that environmental factors influence virus community functions (*e*.*g*., virion particle degradation) and that particle-associated communities may be important drivers of virus activity.

## Introduction

Since the “rediscovery” of the high densities of virus particles in the marine environment [1], viruses are increasingly recognized as key drivers of ecosystem biology and chemistry. In particular, viruses are thought to maintain microbial diversity [2] by constraining abundant cell types in microbial communities and allowing for the division of niches within the marine system [3]. This suggestion has been supported by both strain level laboratory studies [4] and *in silico* modeling of trophic exclusion in the absence of viruses [3]. Lysis of infected cells releases organic carbon and nutrient elements to the residual microbial community, diverting carbon away from higher trophic levels *via* a process known as the “*viral shunt”* [5, 6]. Given the high estimates for virus-mediated cell lysis in the marine environments, this process is likely important in global scale geochemical cycles [7]. Nevertheless, despite the importance of viruses in marine environments, quantitative data on viral dynamics remain rare for broad swaths of the ocean.

Prior estimates suggest viral activity results in the lysis of an estimated ~ 20% of marine heterotrophs daily [5, 8–10]. Virus production assays have been used to estimate rates at which viruses contribute to host mortality and subsequent nutrient release in many aquatic systems [11–13]. Early studies of the environmental constraints on virus dynamics were restricted to coastal regions [13–15], leaving many open-ocean marine environments underrepresented. More recently, studies have explored underrepresented environments, including the deep ocean [16, 17], marine sediments [18, 19], and pelagic ocean regions [20–22]. Despite this increase in spatial coverage, a complete temporal understanding of virus dynamics is still lacking. Studies examining the seasonality of aquatic virus communities have been primarily focused on coastal regions [23, 24].

Observations indicate that drivers of, and constraints on, virus activity vary geographically; therefore major oceanic basins and perhaps even regions within these basins need to be considered independently. For example, in the Sargasso Sea, chlorophyll concentrations and temperature were shown to correlate with both virus abundance and virus production rates; however, this was not seen in the temperate region of the North Atlantic Ocean, the South Pacific Ocean, or the Western Pacific Ocean [20, 22, 25]. In the North Atlantic, virus abundance correlated to cell abundance, a trend not observed in other basin scale studies [22]. In both the North Atlantic and Western Pacific, no previously measured environmental parameters correlated with virus production rates [21, 22].

**Fig 1:**
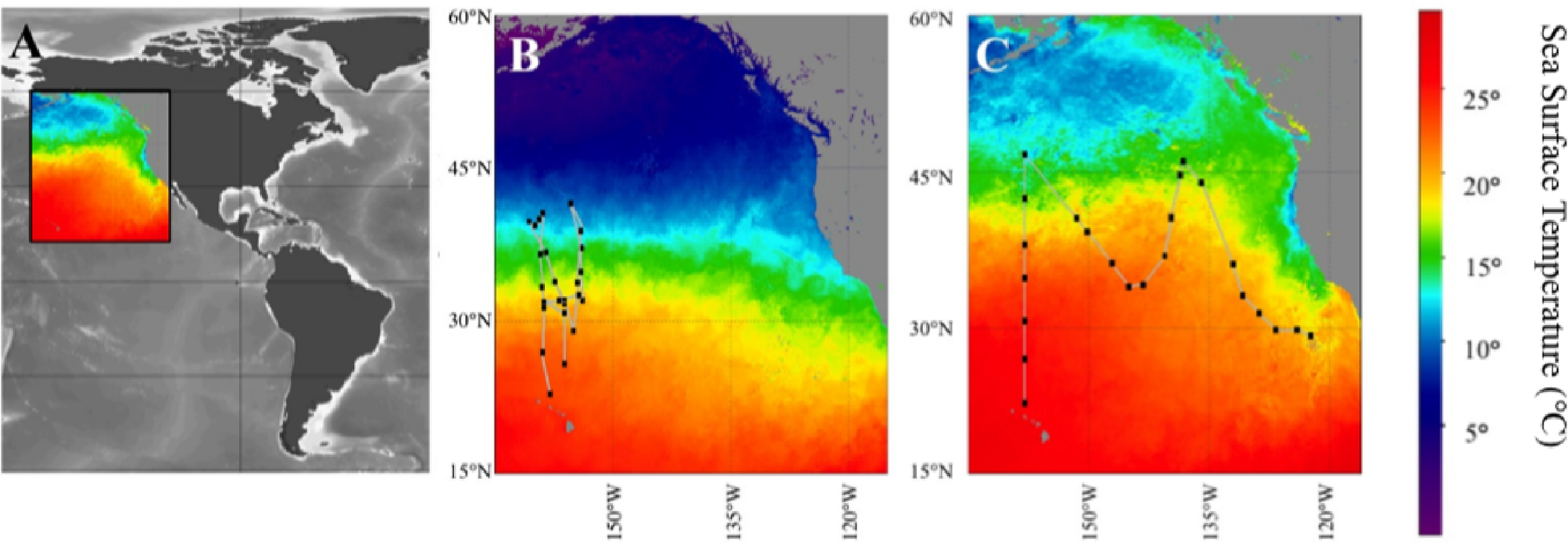
Cruise transects and sampling sites for this study. (A) Color inset indicates location of panels B and C, (B) POWOW 2 (KM1301) cruise transect, (C) POWOW 3 (KM1312) cruise transect. Represented sea surface temperatures based on average values for duration of the cruises as determined by SEA-WIFS (see Materials and Methods for more details).

In the current study, we aimed to address the role host diversity plays, relative to environmental conditions, in shaping virus abundance and production rates. We completed this study in an understudied oligotrophic region, the eastern portion of the North Pacific oceanic basin. This region includes the North Pacific subtropical Gyre (NPSG) (Fig 1A), the Earth’s largest contiguous ecosystem. Viral studies in this region are limited, and primarily focused on sequence-based analysis of virus communities [26, 27]. Our goal was to exploit natural gradients in temperature, pH, and nutrient chemistry in this region to provide insight into factors that may vary seasonally and could play a role in shaping virus dynamics in this region; as many abiotic factors that directly affect viruses likely also influence microbial diversity as well. Understanding the interaction of these factors is vital to the understanding of viral populations and dynamics in this region.

## Materials and methods

### Sample collection and physical parameters

Samples were collected during two cruises aboard the R/V *Kilo Moana*. No specific permissions or permits are required for these locations/activities and research did not involve endangered or protected species. Cruise KM1301 occurred between January 10, 2013 and February 7, 2013 (hereafter referred to as the “winter cruise”). The cruise transect left from Honolulu, HI traversed the NPSG and returned to Honolulu (Fig 1B). KM1312 (hereafter referred to as the “summer cruise”) was conducted between July 1, 2013 and July 28, 2013. This cruise left from Honolulu, HI crossing the NPSG and followed a 19°C isotherm to San Diego, CA (Fig 1C).

As diel periodicity in cellular physiologies of marine communities have been well documented (*i*.*e*., rRNA concentrations [28], chlorophyll concentration [29] and photosynthetic capacity [30]); samples were collected prior to sunrise; (04:00 for winter, 02:00 for summer (local time each day)) in order to capture the community at similar physiological states. All water was collected from the surface (~ 2 m) using a CTD-Niskin rosette. Subsequently, water was transferred into opaque, acid-washed polypropylene carboys (Nalgene) rinsed with water from the same site. Temperature, salinity, *in-situ* chlorophyll a fluorescence, oxygen and PAR within the water column were measured using a CTD Sea-bird 911 equipped with ancillary sensors. Transect maps were created in SeaDas-SeaWiFs Data Analysis System 7.1 [31] using average sea surface temperatures for the duration of the cruise accessed through NASA Sea-viewing Wide Field-of-view Sensor (SeaWiFS) Ocean Color Data (Files A201300012013031.L3m_MO_SST_9 and A20131822013212.L3m_MOSST_9 [accessed on 2015/04/4]. All metadata collected has been uploaded and may be accessed through http://www.bco-dmo.org/project/2237.

### Bacterial abundances, productions, and biomass estimations

A FACSCalibur flow cytometer (Becton Dickinson- Franklin Lakes, NJ, USA) was used to determine bacterioplankton densities as previously described [32]. Briefly, a 488 nm laser was used to measure: inelastic side / forward scatter, green fluorescence, orange fluorescence, and red fluorescence emissions. These measurements were used to differentiate subsets of the bacterioplankton communities. Phytoplankton communities were defined using the red autofluorescence profile (chlorophyll excitation) characteristic of the dominant populations including *Prochlorococcus*, *Synechococcus*, and picoeukaryotes as detailed in Johnson et al. [32]. The total bacterial abundances were determined utilizing a SYBR Green I (Molecular Probes Inc. Eugene, Oregon. United States) staining technique as detailed in Marie et al. [33].

Bacterial production protocols were adapted from Kirchman et al. [34]. Briefly, 20 nM of tritiated leucine was added to triplicate tubes containing 1.7 mL of raw surface seawater. Samples were incubated in the dark at *in-situ* temperatures for 3 h. Killed controls were prepared prior to incubation *via* addition of trichloroacetic acid (TCA) to a final concentration of 1%. Leucine incorporation was stopped via addition of TCA. Cells were then pelleted, rinsed, and dried for 12 h. Samples were resuspended in Ecoscint A (National Diagnostics Atlanta, Georgia. United States) and measured on Packard Bioscience Tri-Carb 2900TR liquid scintillation counter onboard the ship. Measured activity was converted to μg C L^-1^D^-1^ as follows: disintegrations per minute (dpm) of the live samples were corrected with data from killed controls to account for abiotic association of leucine to particles. This value was converted to μCi using the value 2.22x10^6^ dpms per μCi. Following this conversion sample size, incubation time and specific activity of the leucine were accounted. Incorporation of tritiated leucine was converted to moles of carbon through multiplication by a factor of 1.5 [34].

Chlorophyll concentrations were determined by vacuum filtering 100 mL of seawater on 0.22-μm or 0.8-μm polycarbonate filters (Millipore). Chlorophyll was extracted in 100% methanol at -20°C for 24 h [35]. Following extraction, fluorescence of samples was measured with a calibrated Turner Designs 10-AU fluorometer as previously described [36].

### Nutrient analysis

All samples for nutrient measurement were collected in HCl-cleaned, high-density polyethylene bottles (VWR#414004–110) and stored at -80°C until analysis. Subsequent nutrient analysis was performed on technical duplicates of each sample replicate on an Astoria-Pacific A2 autoanalyzer using Certified Reference Materials, (Inorganic Ventures) as follows: Nitrite: QCP-NT; PO_4_ and NO_3_: QCP-NUT-1; SiOH_4_: CGSI1-1.The limit of detection for nutrients is as follows. Phosphorus for both cruises was 0.05 μM. A limit of detection of 0.2 μM SiOH_4_ for samples from KM1301 while 0.1 μM from KM1312. The detection limits for nitrogen species are as follows: the detection limit for nitrite was 0.05 μM for both cruises; and nitrate was 0.1 μM for samples collected on the winter cruise and 0.05 μM for samples from the summer KM1312 cruise.

Samples for ammonium measurements were collected from Niskin bottles as described. Ammonium concentrations were determined using the fluorometric method described in Holmes et al. 1999 [37] and verified using CRMs (Inorganic Ventures: QCP-NUT-1). The limit of detection was 5 nM.

### Virus abundance and production

Virus samples for enumeration were collected from Niskin bottles and fixed with 0.5% (v/v, final concentration) glutaraldehyde and flash frozen in liquid nitrogen for shipping. Upon delivery to the lab, all samples were stored at -80°C until processed. Enumeration of virus particles was performed using a protocol adapted from Ortmann and Suttle [38]. Briefly, 1 mL of sample was vacuum filtered on a 0.02-μm filter Anodisc filter (Whatman). Filters were stained with SYBR green for 20 min, after which excess stain was rinsed from the filter. Viruses were enumerated on a Leica Epifluorescent microscope (Model DM5500 B) using the L5 filter cube at 1000x magnification. Twenty grids or 200 viruses per sample were counted. This protocol is accessible at https://dx.doi.org/10.17504/protocols.io.iptcdnn.

Virus production assays were conducted using the dilution and reoccurrence technique previously described [13,14] and available at https://dx.doi.org/10.17504/protocols.io.dsp6d (Approach 1). Virus-depleted surface water was generated daily using a tangential flow filtration device (Millipore Labscale™ TFF) equipped with a 30kDa filter (Millipore Pellicon XL Filter). Unattached virus particles were reduced in the sample by rinsing the bacterial community three times with virus-free water over a 47 mm diameter 0.2-μm polycarbonate filter (Millipore). Following unattached virus reduction, the bacterial community was resuspended in 500 mL of virus depleted seawater and divided into 3 polycarbonate bottles which were incubated at *in situ* temperature and light levels (screened with neutral density filters to reduce light to ~ 37% ambient) for 12 h. Samples were collected and preserved using 0.5% glutaraldehyde, flash frozen in liquid nitrogen and stored at -80°C until enumeration. For enumeration of virus like particles (VLPs) samples were vacuum filtered onto 0.02-μm pore-size, 20-mm diameter Anodisc filters (Whatman Little Chalfont, Kent, United Kingdom). Filters were stained with SYBR Green I for 30 min in the dark. Slides were preserved with PBS and o-phenylenediamine.

Following staining and preservation, 20 images per slide were acquired using the Leica MM AF Acquisition software within the Leica Application Suite X. Virus-like particles were counted using the Leica MM Analysis Software with thresholds limiting analysis of samples with fewer than 100 virus-like particles per grid or greater than 600 virus-like particles per grid. Samples were enumerated automatically using the thresholds of size greater than 10 pixels and less than 0.55-μm and a border within 5% of a perfect circle. This was compared to manual counts and shown to be statistically similar (data not shown).

Statistical analysis of viral distribution and production was completed in Sigmaplot using Systat functions (ver 12.5). The Shapiro-Wilk test was used to determine the normality of data. If data was non-normally distributed, a Mann-Whitney Rank Sum test was used to determine statistically significant differences between seasons. If data was normally distributed a two tailed t-test was used to determine statistical difference. As a majority of data was non-normally distributed, Spearman correlations were used to determine correlations of factors. Correlations were considered significant if the p-values were less than 0.05.

### Microbial community analyses

Water was collected from the CTD as described above. Cells from 350 mL of seawater were collected onto 47-mm diameter, 0.2-μm nominal pore-size polycarbonate filters (Millipore Billerica, Massachusetts United States) by vacuum filtration. Following collection, samples were placed in 1 mL cryovials and stored in liquid nitrogen for transport back to the lab. After being returned to the laboratory, samples were stored at -80°C until extraction. DNA was extracted using the PowerWater® DNA Isolation Kit (Mo Bio) according to manufacturer’s protocol. The V4 region (*E*. *coli* bases 515–806) of bacterial 16S rRNA genes was amplified and sequenced. Hudson Alpha Genomic Services Laboratory (Huntsville, AL, USA) performed 250-bp paired end sequencing on the Illumina Miseq platform using V2 chemistry. Only reads which reached a Q-score of 30 with no ambiguous bases were used in subsequent analysis. Sequences have been depostited and are available from the NCBI under Bioproject PRJNA394135.

For OTU classification, reads were clustered at 97% similarity and taxonomy was assigned in MOTHUR [39] using the Silva ribosomal database [40]. OTUs identified as being mitochondrial were removed from analysis. Eukaryotic chloroplast DNA was left in libraries as this has been shown to be an effective proxy for eukaryotic phototroph diversity [41]. OTU abundance tables were further analyzed using PRIMER7 [42]. Libraries were normalized based on library size and OTUs were fourth root transformed to achieve normal distribution of counts. A Bray-Curtis resemblance matrix was created, and utilized for multivariate analysis including BEST analysis. To determine the role viruses may play in shaping microbial community structure, a BEST analysis was conducted with all environmental parameters. To determine microbial taxa that may play a role in shaping virus dynamics, a Bray-Curtis resemblance matrix was created including virus abundance and virus production rate. An OTU table was limited to microbial taxa that comprised more than 0.5% of the total bacterial community. Subsequently, a BEST analysis was done to determine which OTUs distribution patterns most closely resembled that of changing virus abundances and production rates. This analysis was done with 500 iterations of stepwise addition of OTUs to determine which were most strongly linked to changing virus abundances and production rates. P-values were determined through 999 permutations.

Analysis of sub-ecotypic diversity of *Prochlorococcus* has been described by Larkin et al. [10]. Briefly, microbial cells were collected from surface water across both transects. DNA was extracted and the internally transcribed spacer region was PCR amplified using *Prochlorococcus* specific primers tRNA_789-F and 23S-R. Amplicon libraries were sequenced using a 454+ GS FLX+Titanium platform (Roche, Basel, Switzerland) and deposited under SRP065205. Identification of environmental parameters correlated to changes in *Prochlorococcus* community structure was conducted in R (R software, v.3.1.2, Vienna, Austria).

## Results

Although the two cruise transects varied geographically within the NPSG, similar physical parameters were observed in surface waters of both winter and summer seasons. Temperatures on the winter cruise ranged from 10–23°C, and in the summer ranged from 11.1–25.5°C; covering largely the same range **(**Shapiro-Wilk P = 0.582, t-test P = 0.06) (Fig 1). This similar temperature range was achieved through sampling temperatures at varying latitudes between seasons. Furthermore when comparing summer and winter, nutrient concentrations including silica (Mann-Whitney P = 0.177), NO_2_ (Mann-Whitney P = 0.596), NO_3_ (Mann-Whitney P = 0.991), NH_4_ (Mann-Whitney P = 0.932), and PO_4_ (Mann-Whitney P = 0.199) were statistically similar.

**Fig 2:**
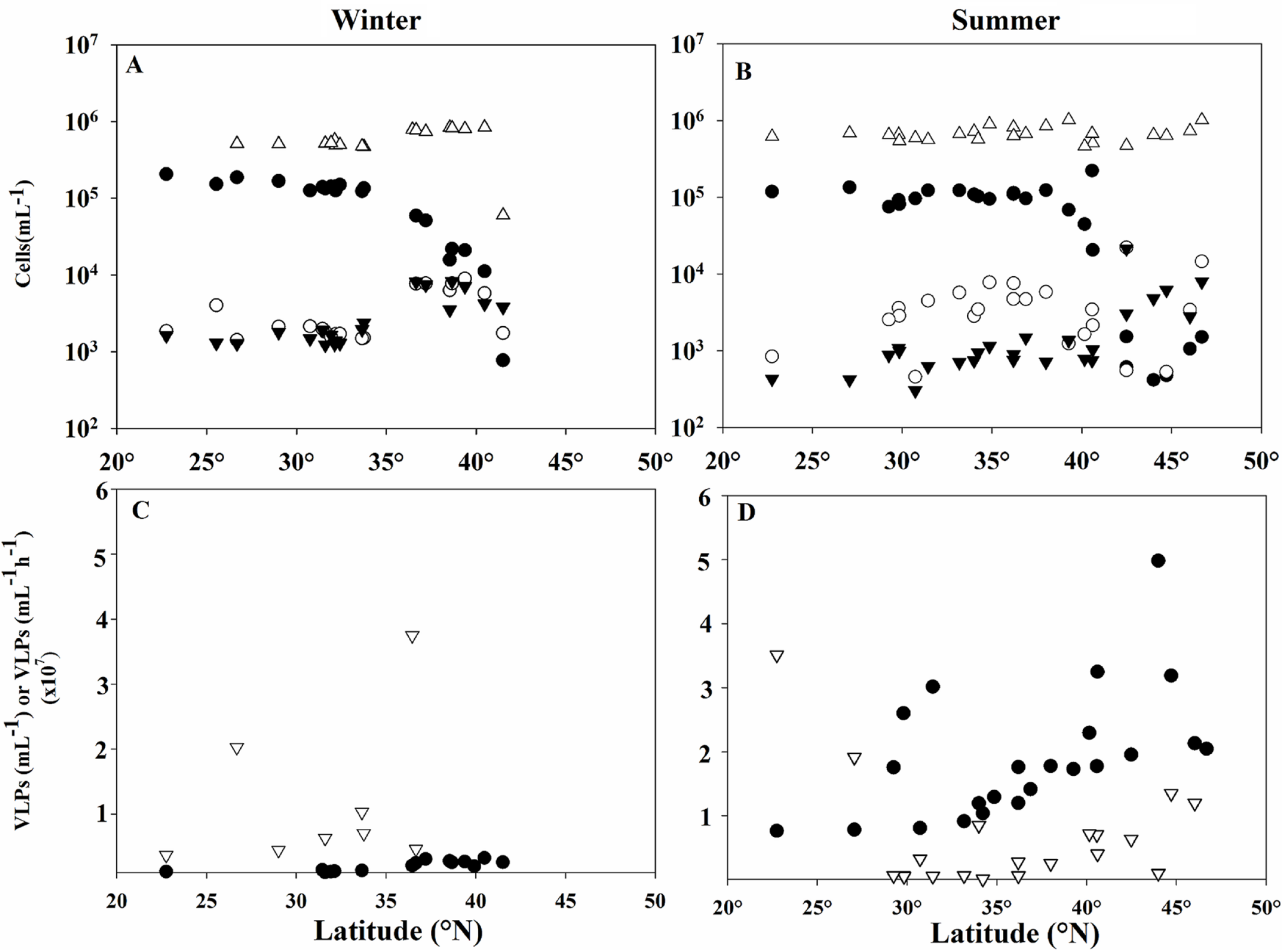
Biological parameters in relationship with latitude. Cell abundances for *Prochlorococcus (*●*)*, *Synechococcus (*○*)*, *Picoeukaryotes (*▼*)*, and total bacterioplankton (including cyanobacteria) (△) across the latitudes investigated in winter (A) and summer (B). Virus abundances (●) and virus production rates (▽) for winter (C) and summer (D).

Total bacterial densities were statistically similar for both cruises, with abundances ranging from 6 x10^5^ to 8.4 x10^5^ cells mL^-1^ in winter and 4.6 x10^5^ to 1.0 x10^6^ in summer (Mann-Whitney P = 0.343). However, the average abundance of *Prochlorococcus* varied between seasons (Mann-Whitney P = 0.022), with populations ranging from 775 to 2 x 10^5^ cells mL^-1^ in winter and 418 to 2.25 x 10^5^ cells mL^-1^ in the summer, in each case with cell density decreasing with increasing latitude. Picoeukaryote densities (Mann-Whitney P = 0.001) were statistically different between cruises as well, with higher abundances measured in the winter. In contrast, *Synechococcus* abundances were not different between cruises (Mann-Whitney P = 0.918), (Fig 2A and 2B).

**Fig 3:**
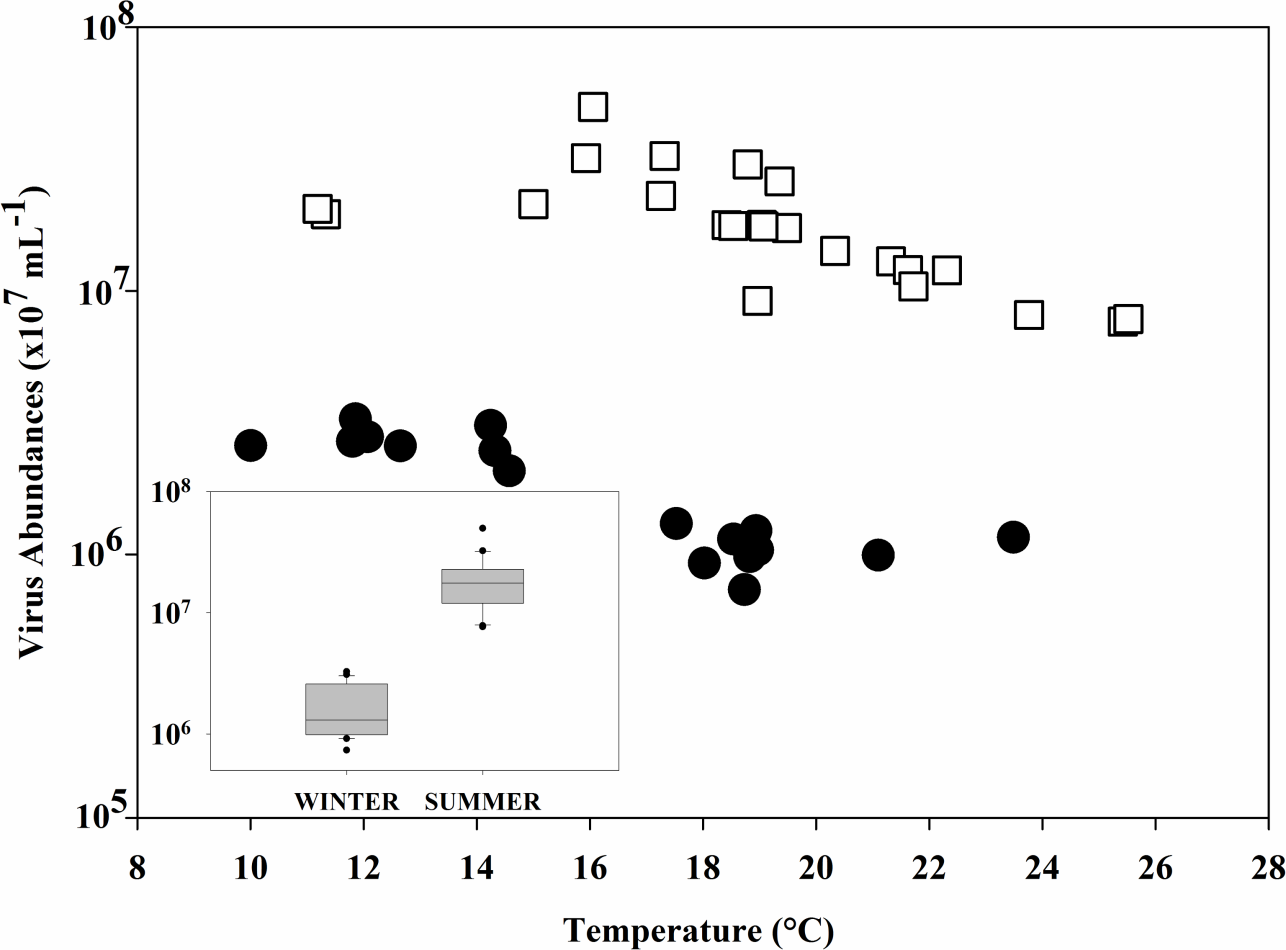
Relationship between temperature and virus abundances. Relationship between virus abundances and temperature for winter (●) and summer (⧠). Inset indicates average virus densities during the winter and summer cruises. * indicates a p-value <0.01.

Average virus abundance was statistically lower in the winter (Mann-Whitney P<0.001), where virus-like particles (VLPs) per milliliter ranged from 7.33 x10^5^ to 3.26 x 10^6^ while in the summer abundances were 7.64 x 10^6^ to 4.98 x 10^7^ (Fig 3). Although the range of production rates were narrower in the summer (1.31 x 10^5^−3.51 x 10^7^ VLPs/mL/h) than in the winter (6.56 x 10^5^ to 1.29 x 10^8^ VLPs/L/d), this difference was not statistically significant (Mann-Whitney, P = 0.095), Fig 2C and 2D.

**Fig 4:**
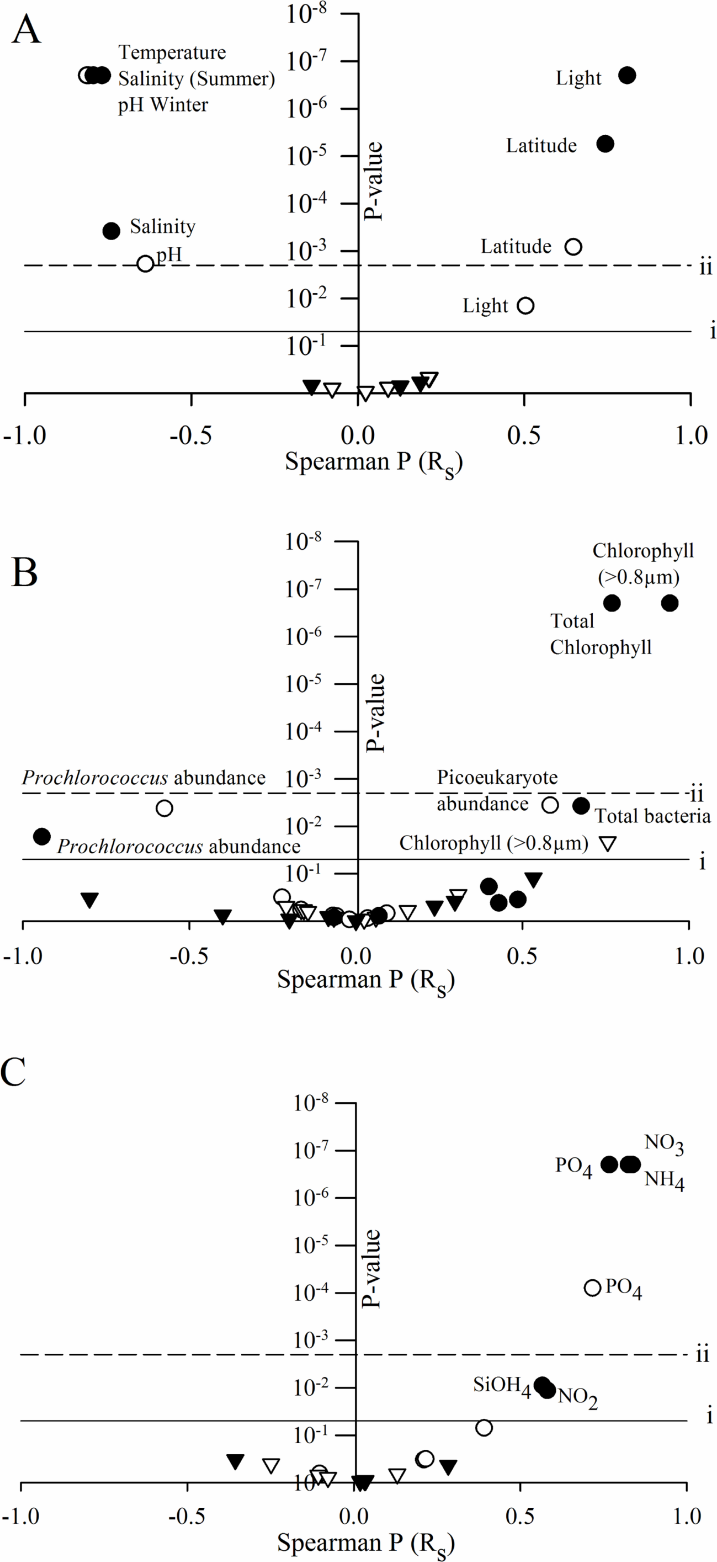
Correlations between virus abundance and productions and environmental parameters. Spearman correlation coefficient shown on the x-axis and p-values indicated on the y-axis. Parameters were separated according to classifications including: physical (A), biological (B) and nutrient(c) data. Circles indicate spearman correlations of virus abundances while virus production correlations are indicated by triangles. Closed symbols = summer samples and open symbols = winter samples. Horizontal dash line (i) shows a p-value of 0.05; solid line (ii) shows a Bonferonni adjusted p-value of 0.002.

Spearman rank correlation analysis was employed to determine factors that related to virus abundance and production rates. Due to strong covariance of abiotic factors and their inextricable links, latitude was used as a generalized descriptor for not only geographical changes, but also these covarying physical parameters. These include covarying factors: temperature (winter: R_s_ = - 0.979, P = < 0.001; summer: R_s_ = -0.804, P = < 0.001), pH (winter: R_s_ = - 0.925, P = < 0.001; summer: R_s_ = -0.804, P = < 0.001), salinity (winter: R_s_ = -0.967, P = < 0.001; summer: R_s_ = -0.778, P = < 0.001), and light (winter: R_s_ = 0.727, P = < 0.001; summer: R_s_ = 0.794, P = < 0.001). None of the factors we measured correlated to virus production rates in the NPSG, with the exception of chlorophyll *a* concentrations in the 0.8-μm fraction of during the summer cruise. Many correlations were seen in relation to viral abundances, a majority of which occurred in both seasons (Fig 4**)**. The virus-like particle distribution in surface waters was strongly correlated to geographical and physical parameters in both seasons, as exhibited by latitude (Fig 4 and S1 Table). Nutrient concentrations of the surface oceans related to virus abundances similarly in both seasons, with virus particle abundances increasing with higher concentrations of most nutrients (the exception being ammonium, which was not correlated to viral abundances in winter).

Several biological parameters correlated with virus abundance independent of cruise season. The density of *Prochlorococcus* cells, as determined by flow cytometry, was negatively correlated to the density of viruses across transects (Fig 4A). Other cyanobacterial populations (*i*.*e*., *Synechococcus*) were correlated to virus abundance only in winter (winter R = 0.549 P = 0.015; summer R = 0.035 P = 0.873), a trend also seen when observing total bacterial cell counts (winter R = 0.676 p = 0.004; summer R = -0.082, p = 0.773). The reverse was seen in an examination of picoeukaryotic density, which was positively associated with viral abundances only in the summer (Fig 4B). Chlorophyll concentrations were the only factor that correlated with virus production rates, and this correlation only occurred in the summer. In winter, only virus abundances correlated to chlorophyll concentrations, possibly indicating a link between microbial primary production and viruses.

To determine whether microbial community diversity influenced virus particle distribution, we explored 16S rRNA gene amplicon libraries collected from surface waters at each station. Alpha diversity metrics (*i*.*e*., OTU richness, Shannon’s Diversity Index, etc.) of sample sites were correlated with viral abundance or production rates, regardless of season. Beta diversity however was linked to viral abundances. Briefly, the SIMPROF analysis within Primer7 determined communities that were statistically indistinguishable from one another. Unsurprisingly, numerous microbial communities clustered into SIMPROF groupings of two or more stations. SIMPROF groups shared similar viral abundances. Subsequently the BEST analysis was used to determine what environmental factors co-vary with microbial community composition. This analysis indicated changes in microbial community structure were most strongly linked to viral abundances and temperature, (R_s_ = 0.157, P = 0.001).

**Fig 5:**
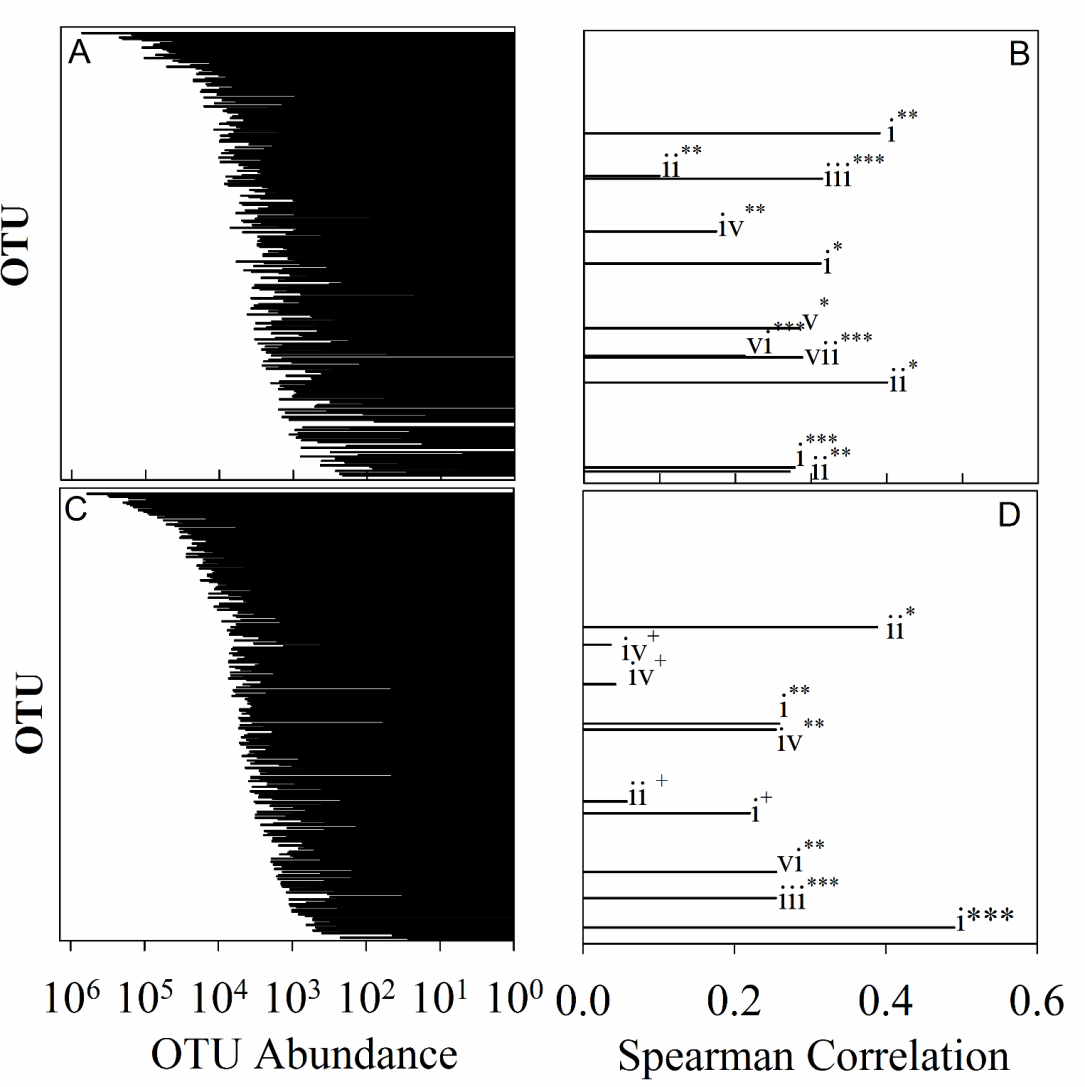
OTU abundance and contribution to variation in viral abundance and production. Plots comparing rank order abundance for individual OTUs and contribution to the proportion of variation in virus abundance and production rates that each OTU contributes. Pairwise data are presented, rank order; contribution to virus dynamics, winter (A; B) and summer (C; D), respectively. Phyla indicated as follows: Chloroplast (i), Proteobacteria (ii), Cyanobacteria (iii), Planctomycetes (iv), Firmicutes (v), Unclassified bacteria (vi), Bacteroidetes (vii). Statistical significance indicated as follows: * = 0.001–0.01, ** = 0.01–0.05, *** = 0.05–0.1, + = > 0.1

As viruses are likely dependent on the composition of the microbial host communities, we asked if certain microbial taxa (OTUs) were linked to changes in virus production or virus abundances in the North Pacific, and if these taxa changed seasonally. Virus abundance and production rates are inextricably linked, therefore we utilized a similarity matrix to determine how “related” sampling locations were based on these two metrics. A BEST analysis was done to determine which microbial taxa were most strongly correlated (spearman) to this similarity matrix. Taxa were limited to those which comprised at least 0.5% of any library. BEST analysis was run stepwise with 500 iterations, and p-values determined through 999 permutations. As viral dynamics have been shown to differ between seasons, this analysis was done for each season separately. Surprisingly, taxa which most strongly correlate to changes in viral dynamics in both seasons are not necessarily the most abundant taxa across the transects, or at a given location. BEST analysis was also used to determine which grouping of OTUs describes the largest variance in virus dynamics. In summer 72% of the variation was described by 14 OTUs (p = 0.001), while in winter 59.2% of the variation was described by 18 OTUs (p = 0.02). (Fig 5). The OTUs which best described virus abundance and production rates changed between seasons with only a single chloroplast signature which was most closely related to *Braarudosphaera bigelowii* shown to be important in both seasons (winter: R_s_ = 0.313, p = 0.02; summer: R_s_ = 0.26, p = 0.036).

Overwhelmingly, the taxa linked to changes in virus concentration and production rates were heterotrophic in nature; 76% and 79% in the winter and summer, respectively (S2 and S3 Tables). In both seasons, OTUs within the phylum Proteobacteria were correlated to virus abundance and production, however, the classes within these differed between seasons. In summer, a majority of OTUs that correlated with virus dynamics belonged to the Alphaproteobacteria phylum. Further, the strongest correlative OTU was an Alphaproteobacterial taxon belonging to the OM75 clade of the family Rhodospirillaceae (R_s_ = 0.389, P = 0.007). This clade is ubiquitous in the world’s oceans. However, there are no cultured representatives of this clade so information on its metabolism is limited. Inferences from distributional patterns indicate it is likely an oligotrophic bacterium with a slow growth cycle [43, 44]. Two other Alphaproteobacteria were also associated with changing virus dynamics in the North Pacific in the summer, an unclassified Rhodospirillales and an uncultured member of the E6AD10 clade. The remainder of the Proteobacteria linked to virus dynamics include an unclassified Gammaproteobacterium as well as a Deltaproteobacterium within the family Oligoflexaceae (S3 Table). In winter, key Proteobacterial taxa included three Gammaproteobacteria. Two of these belonged to the order Oceanspirillales: one Pseudospirillum (R_s_ = 0.1, p = 0.098), and one tentatively assigned to Kangiella (although for this taxon, sufficient permutations could not be achieved to assign a statistical weight within the BEST analysis). The third gamaproteobacteria was an Alteramonadales belonging to the genus *Colwellia* (R_s_ = 0.145, p = 0.217). Other Proteobacterial representatives include an unclassified Rhodobacteraceae (R_s_ = 0.1, p = 0.098), and an unclassified Betaproteobacterium (R_s_ = 0.272, p = 0.04).

The second most represented heterotrophic phylum differed between seasons. In the summer Planctomycetes was determined to be important in differentiating virus abundance and rates of production (3 OTUs identified). Bacteroidetes, with 2 OTUs identified, was the second most identified phylum in winter. In the summer, the abundances of 3 genera belonging to the order Planctomycetaceae contributed to the taxa which correlated to changes in virus dynamics: one *Rubripirellula* (R_s_ = 0.037, p = 0.322), one *Pirellula* (R_s_ = 0.255, p = 0.077), and one belonging to the *FS140-16B-02 marine group* (R_s_ = -0.041, p = -0.563). In winter only a single Planctomycete belonging to the genus *Bythopirellula* (R_s_ = 0.175, p = 0.061) correlated to virus dynamics. In winter 2 OTUs belonging to phylum Bacteroidetes were correlative; one belonging to the family Saprospiraceae (NA), and one belonging to the genus *Ulvibacter* (R_s_ = 0.289, p = 0.042).

Although in both seasons a majority of correlated OTUs were heterotrophic, phototrophic organisms were correlated to virus dynamics as well (S2 and S3 Tables). In winter an OTU assigned to Family 1 of cyanobacteria correlated with changes in virus dynamics between sites (R_s_ = 0.315, p = 0.032); while in summer a member of the nitrogen-fixing cyanobacterial genus *Cyanothece* was the only cyanobacterial OTU correlated to these changes (R_s_ = 0.255, p = 0.063). In our analysis *Prochlorococcus*, was not identified as a taxa driving changes in virus abundance and production across the transects. However, analysis *Prochlorococcus* community structure at the ecotypic level indicates that virus abundance and rates of virus production may be important in shaping the community structure of high light adapted strains of *Prochlorococcus* (R^2^ of 0.58 and 0.54, respectively). Although viral parameters were correlated to sub-ecotypic community structure, viruses had less of an effect on shaping these host communities than the environmental variables we measured (**S1 Fig**). For more in-depth analysis of *Prochlorococcus* community and factors that shape subecotypic diversity of *Prochlorococcus* communities in the North Pacific see Larkin et al. (2016) [10].

## Discussion

In this study we examined basin-wide drivers of virus dynamics (production and abundance) in the North Pacific Ocean. While similar work has been undertaken over the last decade in this region [45], we present this information in the new light of large scale- pelagic seasonal surveys across a temperature gradient, which was further complemented with bacterial diversity information. Moreover, we complemented this effort with measurements of microbial diversity across these stations to assess whether physiochemistry or host diversity was a greater driver of virus particle abundance and production rates.

Global distribution of many viral types has long been theorized [46], and while this has been shown with respect to richness, metaviromic studies indicate virus population structure changes between oceanic provinces [26, 47]. Viruses are distributed passively through ocean currents; as such, local virus community structural changes are likely due to selection by abiotic parameters, as reviewed in Mojica et al. (2014) [48]; but also through presence of the microbial hosts. In our work, temperature was negatively correlated with virus abundance: this is likely due to enhanced decay rates that occur during higher temperatures [49]. Virus abundances correlated positively with both dissolved nitrogen as well as dissolved phosphorus, likely because virus particles make up a large portion of these pools in marine systems [50]. Increased access to nitrogen and phosphorus sources could result in increased primary production, and subsequently higher virus particle abundances in the North Pacific due to increased host abundance. Interestingly negative correlations were seen between dissolved nitrate and reactive phosphorus with virus production rates in the South Pacific. This may be due to differences in microbial community dynamics, as the South Pacific samples were collected during a spring phytoplankton bloom/ bust cycle while the North Pacific Gyre represented a more stable (or non-bloom community) [20].

Given that viruses are obligate parasites, the dynamics of viruses must at least in part, be shaped by the genetic richness and / or diversity of available hosts within the microbial communities. In our study, chlorophyll *a* was highly correlated with virus density, suggesting that photosynthetic members of North Pacific microbial communities are, at least in part, driving virus dynamics. Chlorophyll concentration has been previously shown to correlate strongly to virus abundance in the Beaufort Sea [51]; as well as the Sargasso Sea, in which virus production rate was also a correlate [21]. This may indicate a possible link between the amount of inorganic carbon being fixed and the abundances of virus at a given site, supported by chloroplast DNA correlating to virus abundances.

The abundance of *Prochlorococcus* cells showed a weak, but statistically significant, negative relationship with viral abundances (Fig 4B); this may be due to these cells being lysed by viruses, resulting in a decrease in cell abundance while increasing the occurrence of virus. A secondary explanation however suggests that *Prochlorococcus* cell abundances decreased at lower temperatures [10, 52], in conjunction with a decrease in viral decay (and thus an increase in abundance) at these lower temperatures. Indeed, until a mechanism supporting it is resolved, this correlation must be considered spurious, at best. When sub-ecotype variation in the *Prochlorococcus* community was examined, the high light (HL)-II.4 clade was the only sub-ecotype for which a large proportion of variation in diversity was explained by virus abundance (Data not shown). This clade largely represents the *Prochlorococcus* community which dominates in oligotrophic waters in the summer [10].

The only parameter that viral production rates were correlated to, in this region, was the large phytoplankton fraction (as estimated by the > 0.8 μm fraction chlorophyll concentrations). This preliminarily indicates that large phytoplankton, such as large eukaryotic algae and even picoeukaryotes, could be important drivers of virus production rates in the North Pacific. This may be due to either an indirect relationship, such as their contribution to organic carbon concentrations or their potential role as a platform for attached bacteria. However, we cannot rule out a direct relationship due to their own infection and lysis. To this end, our efforts here may be underestimating the importance of large eukaryotic phytoplankton and archaea in viral dynamics in the North Pacific [41,53]. Indeed we do show several eukaryotic phototrophs to be important (S2–S4 Tables) One taxon which may be important in both seasons, *Braarudospaera bigelowii*, is a marine algae recently shown to harbor nitrogen-fixing endosymbionts [54]. This is particularly interesting as the only cyanobacterial signature determined to correlate to virus dynamics in the North Pacific is a member of the genus *Cyanothece*, a diazotrophic marine cyanobacterium [55]. This may indicate that phototrophic organisms capable of nitrogen fixation could influence viral dynamics.

Unlike other pelagic regions, bacterial production rates in our study were not correlated with viral abundance or viral production rates, further supporting the idea that photosynthetic members of the community may be important drivers in this region. Targeted (16S rRNA gene) metagenomes were sampled in coordination with viral measurements that allowed for the linkage of virus abundance to bacterial community structure. A variety of new hypotheses for how certain members of the bacterial community could drive concentrations of virus in the environment exist: these range from effects of burst size differences to decreased competition due to viral lysis of other bacterial phylotypes [2]. Interestingly, viral production rates did not correlate to overall microbial community structure: this is likely due to the confounding effects of multiple environmental factors (including those influencing virus decay rates).

There were, however, some bacterial members of the microbial community that did correlate to the viral parameters measured, with a majority being heterotrophic in nature (Winter = 76%, Summer = 79%). Most of these OTUs were classified as Proteobacteria, which may indicate that this phylum is an important player in the density and rates at which viruses are being produced in the North Pacific. Furthermore, the OTUs that correlated to the viral parameters we measured changed between seasons, indicating that although Proteobacteria may be important regardless of season, the specific taxa that influence virus abundances and production rates change seasonally. Furthermore, members of the phyla Bacteroidetes and Planctomycetes were important in winter and summer, respectively. Unfortunately, for many of the important OTUs there are very few, if any, cultured representatives, so information on their potential metabolisms are limited. However, many of these taxa have been shown to be disproportionately associated with marine particles, including Alphaproteobacteria, Gammaproteobacteria, Planctomycetacia and Bacteroidetes [53, 56–58].

Although this data must be interpreted carefully, this study indicates a potentially important role for Proteobacteria-infecting viruses in the regeneration of nutrients in the North Pacific. It is important to note that microbial community structure is generally stable in oligotrophic gyres, therefore the most dominant taxa are similar across our transects, thus implying that these taxa may be infected at similar rates across these transects. To this end, while these taxa may be important drivers of abundance and production they will not be identified by this analysis. Instead, we identified taxa whose changing abundances correlated with increased concentration or production of viruses. In our analysis these changes correlated with taxa that are enriched in particle-associated fractions of metagenomes [53, 56–58]. This indicates that in the North Pacific Ocean particle association may lead to increased rates of viral lysis, as proposed by Bettarel et al (2016) [59].

Summer viral abundances were found to be comparable to previous studies in the region (ranging from 1.5x10^6^ to 1.5x10^7^ particles mL^-1^) despite slight differences in seasonal sampling, sample collection methodology and VLP enumeration protocols [45]. Our observations of lower viral abundances in winter relative to summer (Fig 3E) have also been reported in coastal systems of Norway, the Gulf of Mexico, and the Western Pacific ocean, indicating that seasonality of viral abundances is an important consideration in understanding virus dynamics in the global oceans [60, 61]. Although limited, open ocean studies of these dynamics strongly support seasonality in viral distributions as well [57–59]. Parsons et al. (2012) measured a strong seasonality in virioplankton abundances at the Bermuda Atlantic Time-series Study (BATS); with counts peaking in September and being most depleted in January.[62]. Although our temporal coverage is limited, the data suggest this pattern may be also exhibited in the North Pacific. Furthermore, our data suggest that the North Pacific exhibits higher viral abundances in summer than reported in other pelagic regions, with the exception of the Western Pacific [21,22,25]. The ranges of viral abundances in winter were similar to those in the Sargasso Sea (1.4 to 18.9 x10^5^ VLPs mL^-1^ d^-1^) and North Atlantic (8.2 to 28.0 x 10^5^ VLPs mL^-1^ d^-1^) [22].

Our observations support previous findings that the factors that constrain viral dynamics can differ between oceanic basins [21]. Although similar environmental factors correlated to virus abundances in the North Pacific as reported in the Sargasso Sea, North Atlantic, and Western Pacific [22], our data indicate that the suite of factors that affect these dynamics differs. When compared to these regions, the North Pacific shared many of the same correlations with the Sargasso Sea. The North Pacific and the Sargasso Sea have a similar latitude range and both exhibit oligotrophic conditions. Additionally, biotic factors such as chlorophyll concentrations and total bacterial cell abundances were correlated with other pelagic regions.

The observation that factors related to viral abundance vary between regions is unsurprising given that virus particle distribution is likely not influenced by a singular parameter, but likely by environmental interactions. Furthermore, it has been noted that environmental parameters that affect decay rates of viruses (*i*.*e*., UV-B) may vary across different oceanic regions [63]. As such, it is important to note that virus abundance at a given location represents the balance of both production as well as decay/removal rates [48]. Beyond this balance, disconnects in correlations between environmental parameters and abundances are likely due to the treatment of the virus community as one particle type. In actuality, total virus abundances are representative of subsets of viruses that infect different hosts that respond differently to environmental parameters [26]. Therefore, host diversity at least partially drives observations between environmental parameters and virus concentrations and production rates. This study also supports the importance of basin scale investigations of viral dynamics to gain a clearer understanding of factors that may be important in constraining the activity of viruses in ecosystems covering a majority of the Earth’s surface biome.

## Supporting information

S1 Table Spearman correlation values for virus abundance and virus production. Spearman correlation values related to volcano plots in Fig 4. BDL–Below detection limit.
(PDF)

S2 Table OTUs identified by BEST analysis (winter). OTUs Identified by BEST analysis as explaining the most variance in virus abundance and production rates over the winter transect. BEST analysis was conducted stepwise with 500 random restarts; P-values were determined using 999 permutations. OTUs which increased spearman’s rho of >0.01 were included in the analysis.
(PDF)

S3 Table OTUs identified by BEST analysis (summer). OTUs Identified by BEST analysis as explaining the most variance in virus abundance and production rates over the summer transect. BEST analysis was conducted stepwise with 500 random restarts; P-values were determined using 999 permutations. OTUs included must increase spearman’s rho by >0.01 to be identified in the analysis.
(PDF)

S4 Table Taxonomic assignments of chloroplast DNA. Top 50 most abundant OTUs identified as chloroplast DNA in Mothur. A representative sequence was chosen from the OTU and top cultured BLAST hit is reported.
(PDF)

S1 Fig nMDS plot of high light *Prochlorococcus* ecotype diversity as determined by the 16S ITS region. Numbers in parentheses are R^2^ values which indicate the correlation strength between the variable of interest and the distribution of stations in the ordination space.
(PDF)
