# Supplementary material for: Contrasting seasonal drivers of virus abundance and production in the North Pacific Ocean
Source: PLoS One. 2017 Sep 7;12(9):e0184371. doi: 10.1371/journal.pone.0184371 (PMC5589214; doi:10.1371/journal.pone.0184371)
Supplement: S1 Table — Spearman correlation values related to volcano plots in Fig 4. BDL–Below detection limit. (PDF) [file pone.0184371.s001.pdf]

		WINTER				SUMMER			
		Abundance		Production		Abundance		Production	
		r_s	P-value	r_s	P-value	r_s	P-value	r_s	P-value
Physical	Latitude	0.743	< 0.001	- 0.139	0.681	0.647	< 0.001	- 0.077	0.785
	Temperature	-0.794	< 0.001	0.188	0.583	- 0.809	< 0.001	0.024	0.928
	Salinity	0.740	< 0.001	0.127	0.707	- 0.814	< 0.001	0.215	0.469
	pH	-0.768	< 0.001	0.127	0.707	- 0.638	0.002	0.213	0.469
	K_d	0.809	< 0.001	0.188	0.583	0.504	0.014	0.091	0.751
Biological	Viral Production	-0.070	0.844	---	----	-0.165	0.578	----	----
	Prochlorococcus abundance	-0.068	0.001	0.433	0.917	-0.575	<0.001	0.024	0.928
	Synechococcus abundance	0.549	0.015	-0.233	0.520	0.035	0.873	-0.157	0.583
	Total Bacteria	0.676	0.004	0.533	0.124	-0.082	0.773	0.0596	0.832
	Picoeukaryote abundance	0.721	< 0.001	-0.4	0.75	0.583	0.004	-0.208	0.338
	Total Chlorophyll (.22 μm)	0.769	< 0.001	0.236	0.490	-0.058	0.788	0.307	0.279
	Chlorophyll (.8 μm)	0.943	< 0.001	< 0.001	0.983	-0.020	0.934	0.756	0.021
	Secondary Production	0.253	0.390	0.297	0.384	-0.222	0.316	0.155	0.603
Nutrients	PO_4	0.767	< 0.001	-0.356	0.331	0.717	<0.001	0.130	0.656
	$SiOH_4$	0.581	0.011	0.238	0.434	-0.103	0.210	-0.249	0.403
	NO_2	0.566	0.009	BDL	BDL	0.210	0.331	0.035	0.904
	NO_3	0.825	<0.001	0.032	0.919	0.216	0.317	-0.107	0.704
	NH_4	0.836	<0.001	0.018	0.946	0.391	0.071	-0.078	0.785
