# Supplementary material for: Contrasting seasonal drivers of virus abundance and production in the North Pacific Ocean
Source: PLoS One. 2017 Sep 7;12(9):e0184371. doi: 10.1371/journal.pone.0184371 (PMC5589214; doi:10.1371/journal.pone.0184371)
Supplement: S2 Table — OTUs Identified by BEST analysis as explaining the most variance in virus abundance and production rates over the winter transect. BEST analysis was conducted stepwise with 500 random restarts; P-values were determined using 999 permutations. OTUs which increased spearman’s rho of >0.01 were included in the analysis. (PDF) [file pone.0184371.s002.pdf]

OTU	Phyla	Class	Order	Family	Genus	Spearman Rho	P-value
**67**	Cyanobacteria	Chloroplast			Uncultured Picoeukaryote	0.391	0.001
**96**	Proteobacteria	Alphaproteobacteria	Rhodobacterales	Rhodobacteraceae	Unclassified	0.1	0.098
**98**	Cyanobacteria	Cyanobacteria	SubsectionI	FamilyI	Marine Group	0.315	0.032
**137**	Planctomycetes	Planctomycetacia	Planctomycetales	Planctomycetaceae	Bythopirellula	0.175	0.061
**162**	Cyanobacteria	Chloroplast			*Braarudospaera bigelowii* Chloroplast	0.313	0.02
**191**	Proteobacteria	Gammaproteobacteria	Oceanospirillales	Alcanivoracaceae	Kangiella	NA	NA
**216**	Firmicutes	Bacilli	Bacillales		Unclassified	0.285	0.01
**241**	Unclassified Bacteria				Unclassified	0.213	0.092
**243**	Bacteroidetes	Flavobacteriia	Flavobacteriales	Flavobacteriaceae	Ulvibacter	0.289	0.042
**253**	Unclassified Bacteria	Unclassified Bacteria	Unclassified Bacteria	Unclassified Bacteria	Unclassified	0.136	0.225
**282**	Proteobacteria	Gammaproteobacteria	Oceanospirillales	Oceanospirillaceae	Pseudospirillum	0.401	0.007
**396**	Unclassified Bacteria					-0.068	0.557
**400**	Chloroflexi	SAR202 clade			Unclassified	0.041	0.27
**407**	Bacteroidetes	Sphingobacteriia	Sphingobacteriales	Saprospiraceae	Unclassified	Na	NA
**548**	Cyanobacteria	Chloroplast			Uncultured Eukaryote Chloroplast DNA	0.279	0.053
**611**	Proteobacteria	Betaproteobacteria			Unclassified	0.272	0.04
**700**	Euryarchaeota	Thermoplasmata	Thermoplasmatales	Marine_Group_II	Unclassified	NA	NA
**845**	Unclassified Bacteria				Unclassified	NA	NA
**887**	Proteobacteria	Gammaproteobacteria	Alteromonadales	Colwelliaceae	Colwellia	0.145	0.217
					**OVERALL**	**0.592**	**0.02**
