# Supplementary material for: Contrasting seasonal drivers of virus abundance and production in the North Pacific Ocean
Source: PLoS One. 2017 Sep 7;12(9):e0184371. doi: 10.1371/journal.pone.0184371 (PMC5589214; doi:10.1371/journal.pone.0184371)
Supplement: S3 Table — OTUs Identified by BEST analysis as explaining the most variance in virus abundance and production rates over the summer transect. BEST analysis was conducted stepwise with 500 random restarts; P-values were determined using 999 permutations. OTUs included must increase spearman’s rho by >0.01 to be identified in the analysis. (PDF) [file pone.0184371.s003.pdf]

OTU	Phyla	Class	Order	Family	Genus	Spearman Rho	P-value
**90**	Proteobacteria	Alphaproteobacteria	Rhodospirillales	Rhodospirillaceae	*OM75 clade*	0.389	0.007
**102**	Planctomycetes	Planctomycetacia	Planctomycetales	Planctomycetaceae	*Rubripirellula*	0.037	0.322
**126**	Planctomycetes	Phycisphaerae	Phycisphaerales	Phycisphaeraceae	*FS140-16B-02 marine group*	-0.041	.0.563
**131**	Bacteroidetes	Sphingobacteriia	Sphingobacteriales	Sphingobacteriaceae	*Pedobacter*	0.043	0.344
**162**	Cyanobacteria	Chloroplast			*Braarudospaera bigelowii* Chloroplast	0.26	0.036
**166**	Planctomycetes	Planctomycetacia	Planctomycetales	Planctomycetaceae	*Pirellula*	0.255	0.077
**219**	Proteobacteria	Alphaproteobacteria	Rhodospirillales		Unclassified	NA	NA
**227**	Proteobacteria	Gammaproteobacteria			Unclassified	0.058	0.268
**238**	Cyanobacteria	Chloroplast			Uncultured Eukaryote Chloroplast DNA	0.221	0.136
**266**					Unclassified	0	0.709
**308**	Proteobacteria	Deltaproteobacteria	Oligoflexales	Oligoflexaceae	Unclassified	0	0.684
**323**	Bacteroidetes	Flavobacteriia	Flavobacteriales	Flavobacteriaceae	*Joostella*	0.256	0.034
**399**	Proteobacteria	Alphaproteobacteria	E6aD10		Unclassified	NA	NA
**447**	Cyanobacteria	Cyanobacteria	Subsection I	FamilyI	*Cyanothece*	0.255	0.063
**548**	Cyanobacteria	Chloroplast			Uncultured Eukaryote Chloroplast DNA	0.491	0.001
					**Overall**	**0.72**	**0.001**
