# Supplementary material for: Contrasting seasonal drivers of virus abundance and production in the North Pacific Ocean
Source: PLoS One. 2017 Sep 7;12(9):e0184371. doi: 10.1371/journal.pone.0184371 (PMC5589214; doi:10.1371/journal.pone.0184371)
Supplement: S4 Table — Top 50 most abundant OTUs identified as chloroplast DNA in Mothur. A representative sequence was chosen from the OTU and top cultured BLAST hit is reported. (PDF) [file pone.0184371.s004.pdf]

	Taxonomy Name	E-Value	Sim	Accession
**Otu00023**	*Helicopedinella sp. RCC2284*	8.44E-115	100.00%	LN735338.3
**Otu00036**	*Bacillariophyta sp. 867-32*	2.84E-118	99.10%	KP792487.1
**Otu00054**	*Emiliania huxleyi*	2.84E-118	99.10%	JN022705.1
**Otu00062**	Uncultured Marine Eukaryote	5.22E-120	99.60%	KX938088.1
**Otu00064**	*Phaeocystis globosa*	5.90E-116	99.10%	KC900889.1
**Otu00067**	Uncultured Picoeukaryote	4.60E-113	97.80%	JX291751.1
**Otu00079**	Uncultured Dictyochophyte	1.55E-116	98.70%	EF052136.1
**Otu00086**	*Virgulinella fragilis*	1.55E-116	98.70%	JN207213.1
**Otu00097**	Uncultured Marine Eukaryote	2.84E-118	99.10%	KX935068.1
**Otu00100**	*Planoglabratella opercularis*	1.37E-109	97.00%	KP792467.1
**Otu00113**	*Florenciella parvula*	1.55E-116	99.10%	LN735274.2
**Otu00116**	Uncultured Chrysophyte	2.84E-118	99.10%	EF052163.1
**Otu00120**	Uncultured Marine Eukaryote	8.44E-115	98.30%	KX937565.1
**Otu00158**	Uncultured Haptophyte	8.44E-115	98.30%	EF052065.1
**Otu00162**	*Braarudosphaera bigelowii*	2.84E-118	99.10%	AB847986.2
**Otu00218**	Uncultured Chrysophyte	2.84E-118	99.10%	EF052251.1
**Otu00234**	*Asterionella formosa*	4.06E-106	96.10%	KC509519.1
**Otu00238**	*Prasinophyceae sp. RCC999*	1.75E-112	98.30%	LN735517.3
**Otu00242**	*Proboscia inermis*	2.79E-96	94.40%	FJ002201.1
**Otu00255**	Uncultured Marine Eukaryote	3.55E-110	97.80%	KX937647.1
**Otu00260**	Uncultured Marine Eukaryote	5.22E-120	99.60%	KX938009.1
**Otu00268**	Uncultured Marine Eukaryote	5.82E-116	100.00%	KX938202.1
**Otu00270**	Eukaryote SCGC AAA074-P04	2.51E-111	97.40%	JF488733.1
**Otu00276**	*Virgulinella fragilis*	4.60E-113	97.80%	KP792491.1
**Otu00284**	*Ralstonia solanacearum*	2.51E-111	97.40%	CP011998.1
**Otu00319**	Uncultured Marine Eukaryote	2.21E-104	96.10%	KX938047.1
**Otu00335**	*Rhizosolenia imbricata*	4.60E-113	97.80%	KJ958482.1
**Otu00343**	*Proboscia indica*	8.44E-115	98.30%	FJ002241.1
**Otu00344**	Uncultured Marine Eukaryote	1.55E-116	98.70%	KX937668.1
**Otu00364**	*Syracosphaera pulchra*	1.20E-102	96.00%	LN735258.2
**Otu00366**	*Calcidiscus quadriperforatus*	2.51E-111	97.40%	LN735221.2
**Otu00370**	Uncultured Chrysophyte	3.16E-103	96.10%	EF052251.1
**Otu00379**	*Actaea racemosa*	1.07E-117	99.60%	KY085920.1
**Otu00395**	*Teleaulax amphioxeia*	4.62E-113	99.10%	KX816862.1
**Otu00423**	Uncultured Diatom	1.21E-102	95.30%	KX938043.1
**Otu00494**	*Virgulinella fragilis*	2.51E-111	97.40%	JN207220.1
**Otu00502**	Uncultured Chrysophyte	5.22E-120	99.60%	KX938184.1
**Otu00530**	*Chaetoceros calcitrans*	8.44E-115	98.30%	LN735292.2
**Otu00531**	*Thalassionema sp. CCMP1100*	4.06E-106	96.10%	FJ002197.1
**Otu00534**	Uncultured marine eukaryote	2.84E-118	99.10%	KX934876.1
**Otu00548**	Eukaryote SCGC AAA074-K22	8.44E-115	98.30%	JF488711.1
**Otu00549**	*Pelagomonas sp. RCC986*	1.21E-113	100.00%	LN735511.3
**Otu00565**	*Thalassionema sp. CCMP1100*	1.37E-109	97.00%	FJ002197.1
**Otu00573**	*Planoglabratella opercularis*	4.60E-113	97.80%	KP792480.1
**Otu00581**	Uncultured Phototrophic Eukaryote	1.55E-116	98.70%	KP792480.1

**Otu00585**	Uncultured Marine Eukaryote	4.60E-113	97.80%	KX938161.1
**Otu00602**	*Rhizosolenia imbricata*	2.21E-104	95.70%	KJ958482.1
**Otu00624**	Uncultured Marine Eukaryote	1.55E-116	98.70%	KX937932.1
**Otu00636**	Helicosphaera carteri	8.44E-115	98.30%	LN735234.2
