# Supplementary figures and images for: Contrasting seasonal drivers of virus abundance and production in the North Pacific Ocean
Source: PLoS One. 2017 Sep 7;12(9):e0184371. doi: 10.1371/journal.pone.0184371 (PMC5589214; doi:10.1371/journal.pone.0184371)

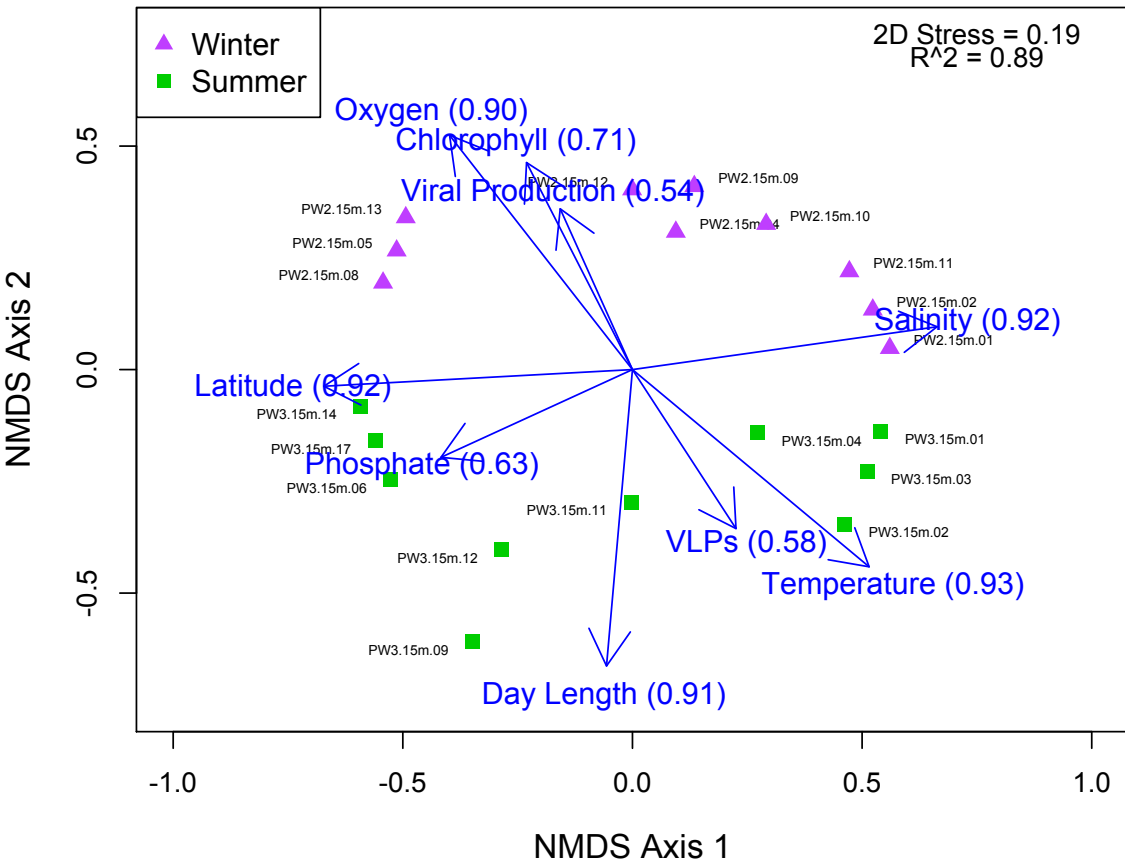

Supplement: S1 Fig — Numbers in parentheses are R2 values which indicate the correlation strength between the variable of interest and the distribution of stations in the ordination space. (PDF) [file pone.0184371.s005.pdf]
